# Virtual scale function of gastrointestinal endoscopy for accurate polyp size estimation in real-time: a preliminary study

**DOI:** 10.1117/1.JBO.26.9.096002

**Published:** 2021-09-01

**Authors:** Masato Yoshioka, Yuichi Sakaguchi, Daisuke Utsunomiya, Shinichiro Sonoda, Takeichi Tatsuta, Satoshi Ozawa, Yuichi Teramura, Keita Harada, Hideaki Kinugasa, Hiroyuki Okada

**Affiliations:** a FUJIFILM Corporation, Tokyo, Japan; b Okayama University Graduate School of Medicine, Dentistry and Pharmaceutical Sciences, Department of Gastroenterology and Hepatology, Okayama, Japan

**Keywords:** endoscopy, laser applications, image processing, real-time imaging, size estimation, virtual scale

## Abstract

**Significance:** Polyp size is important for selecting the surveillance interval or treatment policy. Nevertheless, it is challenging to accurately estimate the polyp size during endoscopy. An easy and cost-effective function to assist in polyp size estimation is required.

**Aim:** To propose a virtual scale function for endoscopy and evaluate its performance and expected accuracy.

**Approach:** An adaptive virtual scale behavior was demonstrated. The measurement error of the virtual scale along the distance between the tip of the endoscope and the object plane was evaluated using graph paper. The accuracy of polyp size estimation by an expert endoscopist was compared with the accuracy of the biopsy forceps method using phantom images.

**Results:** The measurement errors of the virtual scale were ≤0.7  mm when the distance to the graph paper, which faced the tip of the endoscope, varied from 4 to 30 mm. The accuracy with the virtual scale was significantly higher than that obtained with biopsy forceps (5.3±5.5% versus 11.9±9.4%, P<0.001).

**Conclusions:** The virtual scale function, which operates in real-time without any additional device, can be used to estimate polyp sizes easily and accurately with endoscopy.

## 1 Introduction

Polyp size is an important risk factor for colorectal cancer. Many guidelines or reports of colonoscopy make recommendations or provide decision trees according to the size of the detected polyp/adenoma. For example, the recommendation of the colonoscopy surveillance interval after polypectomy is divided based on whether the adenoma size is ≥10  mm or less in some guidelines.^1^^–^^3^ According to the preservation and incorporation of valuable endoscopic innovations in real-time endoscopic assessments of the histology of diminutive colorectal polyps proposed by the American Society of Gastrointestinal Endoscopy,^4^ diminutive lesions smaller than 5 mm should be targeted for resection and discarded because they are associated with low rates of invasive cancer and advanced histology such as villous adenomas and highly atypical adenomas. Regarding the treatment policy for colorectal lesions, polyps of sizes ≤20  mm are assessed to determine whether they can be resected at once with a snare, and the treatment method is selected (either endoscopic mucosal resection or endoscopic submucosal dissection).^5^

Polyp size is important information for endoscopists; however, it is challenging to estimate it accurately during the procedure. Chaptini et al. reported that only 48% of polyp size estimates were within 20% of the correct value according to a web-based video survey conducted with the participation of 873 endoscopists.^6^ Pham et al.^7^ retrospectively investigated the sizes of 189 adenomatous polyps and identified incorrectly measured adenomatous polyps, defined as polyps with size variations >33%, from the measurement data collected from resected specimens with a ruler post-fixation, amounting to 56.6% of the studied cases. The size of an object on a monitor changes depending on the distance between the tip of the endoscope and the object; thus, it is challenging to determine the exact size only based on two-dimensional endoscopic images. Furthermore, the displayed image is distorted so that the corresponding size on the image against the actual size is different when the central and peripheral parts of the image are compared. This is because the objective lens of the image sensor of the endoscope has a fisheye-like structure to observe wide field-of-views in luminal structures. The European Society of Gastrointestinal Endoscopy indicated the measurement bias associated with polyp sizes and recommended the use of a standardized polyp size measurement in the guidelines for post-polypectomy colonoscopy surveillance.^1^ It is known that the measurement bias can be reduced using reference standards, such as open biopsy forceps or snares.^8^^,^^9^ Some additional devices, such as graduated injection needles,^10^ ruler snares,^11^ calibrated hood,^12^ virtual tape measures,^13^ or structured light laser probes that project lattice patterns on the object,^14^ have been proposed to improve accuracy. These devices are effective for estimating the polyp size accurately. However, using additional devices only for polyp size estimation is cumbersome and not effective in terms of procedural time and cost. An easy and cost-effective function used to assist polyp size estimation is desired.

In this study, we developed a virtual scale function for an endoscope, which adaptively changes the length in real-time according to the distance to the object to be measured for accurate polyp size estimation during the procedure. As a preliminary study to understand the fundamental performance and expected accuracy of the virtual scale function, the accuracy of the adaptive movement and accuracy of polyp size estimation were evaluated using phantom images.

## 2 Materials and Method

### 2.1 Principle of the Virtual Scale Function

**Fig. 1:**
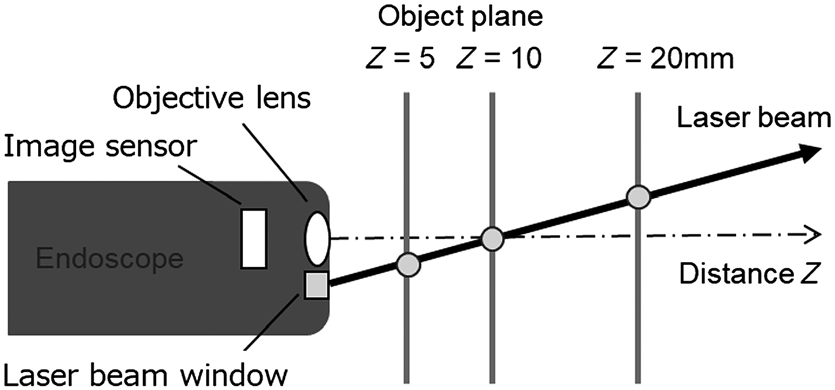
Schematic of the distance estimation function.

The principle of distance estimation to the object to adaptively change the length of the virtual scale was based on the triangulation method. A schematic of the tip of the endoscope is shown in Fig. 1(a). The endoscope has a window at the tip to emit the red laser beam diagonally so that the position of the laser beam on the object changes according to the distance between the object and the tip of the endoscope. The output power of the red laser was weak and did not have an effect on the mucosa. When the image sensor of the endoscope detects the laser spot, the distance from the tip of the endoscope to the object illuminated by the laser is calculated from the position of the laser spot.

An endoscope prototype with a built-in red laser with a diameter of 12.8 mm was implemented for colonoscopy. The endoscope is part of the ELUXEO system (FUJIFILM Co., Tokyo, Japan) comprising a VP-7000 processor and BL-7000 light source. It supports image-enhanced functions, such as linked color imaging (LCI) or blue-light imaging (BLI), which are effective for the detection and characterization of polyps.^15^^–^^19^ The image signal from the processor was input to a personal computer EX-1 (FUJIFILM Co., Tokyo, Japan), and dedicated software was installed to estimate the distance in real-time. The output of the image signal was displayed on a monitor with a suitable representation of the virtual scale. The laser beam spot was located at the left end of the virtual scale. The virtual scale contained markings at 5, 10, and 20 mm, as shown in Fig. 4. The length of the virtual scale and the steps were adaptively changed according to the distance to the object, which was calculated from the position of the laser spot. The red laser and the virtual scale can be switched on and off by a button on the handle of the endoscope. This virtual scale function can operate in the conventional white light imaging mode as well as in the LCI mode. When the operator changes to the BLI mode, the virtual scale is automatically switched off.

### 2.2 Evaluation of Fundamental Performance

The length of the virtual scale is calculated from the position of the laser spot assuming that the object is on the plane facing the tip of the endoscope. A measurement error will occur according to the distance of the object and the tilt angle along the direction of the virtual scale bar.

To evaluate the fundamental performance, the measurement error of the adaptive virtual scale was evaluated using graph paper with ruled lines (0.5-mm increments) as the measurement object. From the endoscopic image of the graph paper with the virtual scale, the actual length between the position indicated by the left end and the 5, 10, or 20 mm marks of the virtual scale was read off from the graph paper. The range of the measurable actual length in this graph paper was ≤30  mm. The measurement error was defined as the difference of the actual length from the corresponding step of the virtual scale. First, the measurement error was read off when the distance to the graph paper, which faced the tip of the endoscope, varied from 4 to 30 mm. The maximum measurement error, mean ± standard deviation of the measurement error, and the standardized measurement accuracy, defined as the absolute value of the measurement error divided by the actual size multiplied by 100%, were within the measurement range. The measurement error was read off when the graph paper was tilted left or right by 30 deg with respect to the vertical centerline of the endoscopic image when the graph paper was placed at distances of 10, 20, or 30 mm. The origin of the tilt angle was defined as the reference point at which the graph paper faces the endoscope. It was defined as positive when the left side of the endoscope image moved to the front and negative when the right side moved to the front.

### 2.3 Image-Based Estimation of the Polyp Size

To evaluate the accuracy of this virtual scale function, the method used to estimate polyp sizes by expert endoscopists, and their comparisons to the open biopsy forceps method were demonstrated based on the use of phantom images. Silicone pseudo-polyps were attached to the colon model LM-107 (Koken Co., Ltd., Tokyo, Japan). In total, 33 silicon pseudo-polyps with diameters in the range of 2.5 to 28 mm, 21 hemispherical types, six spherical types, and two pedunculated types were studied. Each size was measured with a caliper (unit of 0.1 mm). Endoscopic images were acquired at randomly set distances in the range of 4 to 30 mm from the polyp to the tip of the endoscope for each polyp. The position of the endoscope against the polyp was also randomly set to acquire the view in which the polyp was visualized anteriorly as well as the scene in which the polyp was visualized on the side to emulate actual clinical practice. Each polyp was imaged with the virtual scale or with the biopsy forceps (Radial Jaw4 Jumbo, Boston Scientific, Marlborough, Massachusetts), which had a maximum width of 9 mm in the open state. When images were acquired with biopsy forceps, the forceps were fully opened and pressed against the polyp. When images were acquired with a virtual scale, the spot of the red laser, that is, the left edge of the virtual scale, was aligned with the left edge of the polyp. In total, 33 patterns of images were prepared for biopsy forceps and virtual scale tests.

The endoscopists estimated the sizes of the polyps from the images. As a preliminary preparation for the test, the evaluators were briefed on the principles, directions, and precautions of the virtual scale function. Subsequently, the test method was explained, and four images, which depicted pseudo-polyps with the sizes of 5, 10, 15, or 20 mm, as measured with the biopsy forceps or the virtual scale method, were presented. These image examples were not used in the subsequent tests. The polyp size estimation test was conducted by displaying the polyp images on the endoscopic monitor without any information regarding the actual polyp size. In total, 33 images with the biopsy forceps method were presented first followed by another set of 33 images with the virtual scale method. Within each image set, the order of images was random. Evaluators estimated the polyp size from a displayed image with reference to the biopsy forceps or virtual scale and then filled the answer sheet.

For the analysis of the measurement accuracy, the measurement error (defined as the difference of the actual and estimated sizes), and standardized measurement accuracy (defined as the absolute value of measurement error divided by the actual size multiplied by 100%) were compared between the forceps and virtual scale sets. Correct estimation is defined as when the absolute value of the measurement error <1  mm. Based on this, the numbers and rates of correct estimation, overestimation, and underestimation were analyzed. Continuous data are presented as the mean ± standard deviation. Categorical data are presented as numbers or rates. The difference between biopsy forceps and the virtual scale in categorical data was analyzed using the Fischer’s exact test. The differences in continuous data acquired from the same polyp were analyzed using a paired t- or Wilcoxon signed-rank test. Statistical significance was set at P<0.05. Statistical analysis was performed with R (version 3.6.1, R Foundation for Statistical Computing, Vienna, Austria).

## 3 Results

### 3.1 Demonstration of the Real-Time Adaptive Virtual Scale

**Fig. 2:**
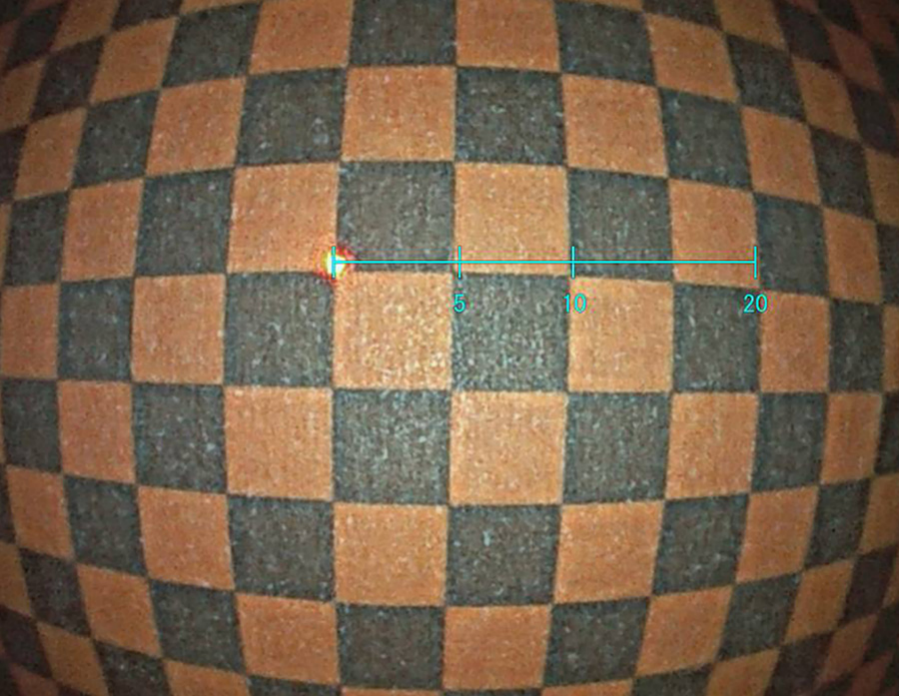
Demonstration of the virtual scale function (Video 1, MPEG, 12 MB [URL: https://doi.org/10.1117/1.JBO.26.9.096002.1]).

The virtual scale behavior, which changes the size on the monitor in real-time according to the distance to the object, was demonstrated (Fig. 2) using a chart sheet and pseudo-polyps. When the operator pushed the button on the handle of the endoscope, the red laser was on and the virtual scale was indicated as a sky-blue bar, which appeared on the monitor. The real-time behavior confirmed that the virtual scale moved and changed the length along with the red laser spot according to the distance to the object. The measurable distance range was from 4 to 30 mm. When the image display range was <20  mm, the total length of the scale was displayed up to 10 mm, and when the image display range was <10  mm, the total length of the scale was up to 5 mm. When the laser spot was not detected or the distance was outside the effective range, an error occurred, and the virtual scale turned yellow.

In clinical practice, when an endoscopist points the tip of the endoscope in front of the object and arranges the spot of the laser beam at the left end of the object, the endoscopist can compare the size of the object with the virtual scale overlaid on the object without any other additional device.

### 3.2 Evaluation of Fundamental Performance

**Fig. 3:**
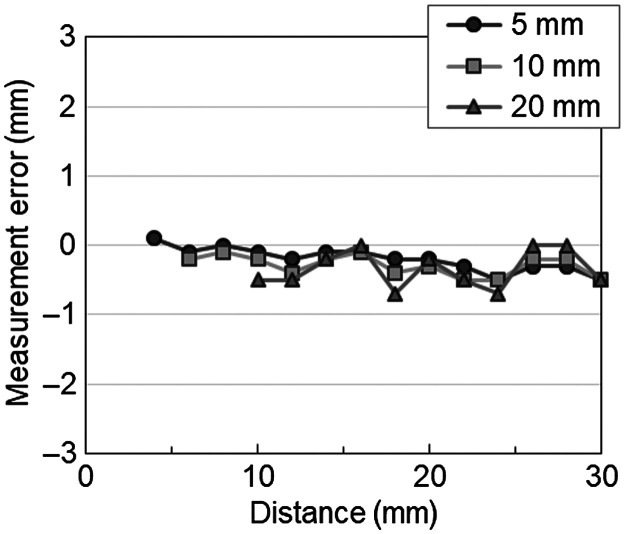
Measurement errors obtained by reading the lengths from the graph paper (at 5, 10, and 20 mm steps of the virtual scale) from the endoscope image when the distance to the graph paper, which faced the tip of the endoscope, varied from 4 mm to 30 mm.

Measurement errors estimated (for 5, 10, and 20 mm steps of the virtual scale) when the distance to the graph paper faced the tip of the endoscope varied from 4 to 30 mm, as shown in Fig. 3. The maximum measurement errors (and the standardized measurement accuracies) were 0.5 mm (9.1%), 0.5 mm (4.8%), and 0.7 mm (3.4%), and the mean ± standard deviations were −0.2±0.2  mm (4±2.6%), −0.3±0.1  mm (2.8±1.4%), and −0.3±0.3  mm (1.7±1.3%), respectively.

**Fig. 4:**
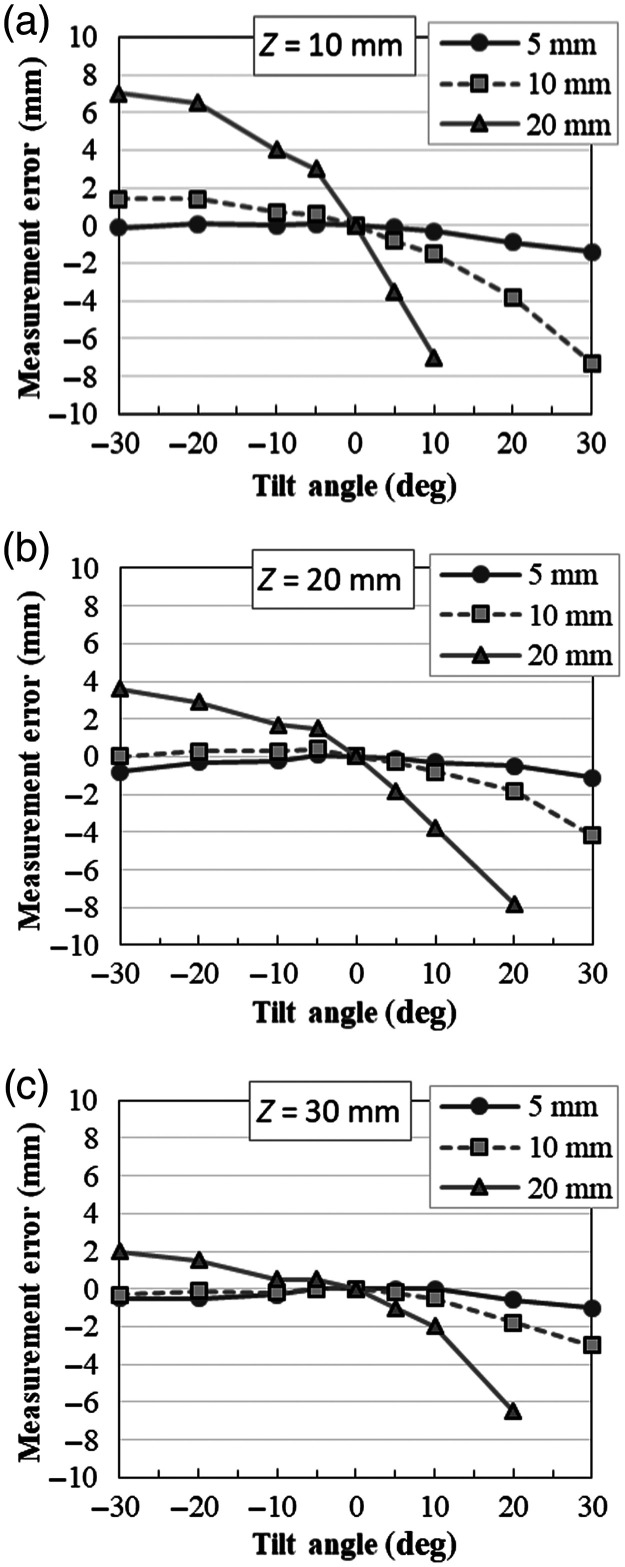
Measurement errors obtained by reading the lengths of the polyps from the graph paper (step intervals of 5, 10, and 20 mm of the virtual scale on the endoscope images) in instances at which the graph paper was tilted left or right by 30 deg. The distances Z to the graph paper were (a) 10, (b) 20, and (c) 30 mm.

**Fig. 5:**
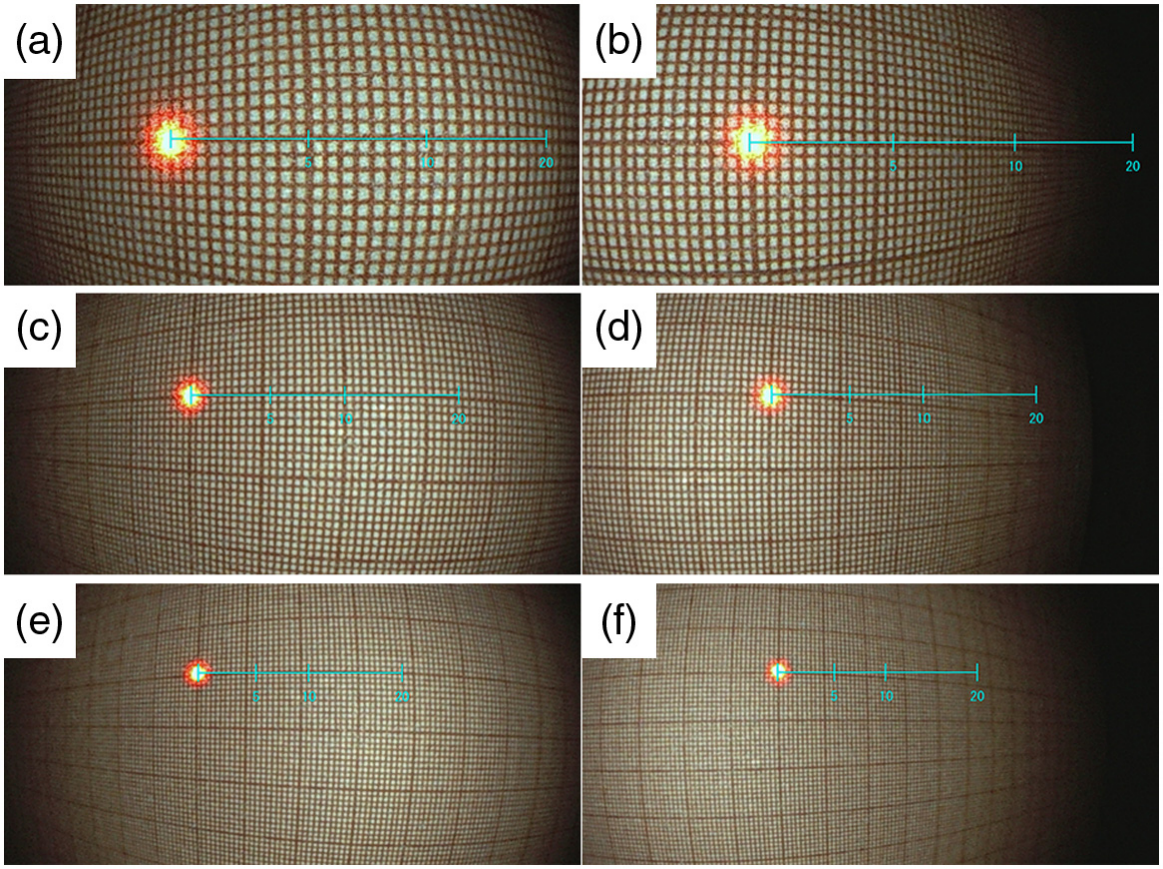
Endoscopic images of the graph paper (virtual scale). The distances and tilt angles of the graph paper were (a) 10 mm, −20  deg, (b) 10 mm, +20  deg, (c) 20 mm, −20  deg, (d) 20 mm, +20  deg, (e) 30 mm, +20  deg, and (f) 30 mm, −20  deg.

The measurement errors when the graph paper was placed at the distances of 10, 20, or 30 mm and was tilted left or right by 30 deg with respect to the vertical centerline of the endoscopic image, are shown in Fig. 4. As the tilt angle and the scale increased, the error also increased. In particular, when the tilt angle was positive, that is, the left side moved to the front, a large error occurred and resulted in an underestimation. The observation distance also affected the measurement error when the graph paper was tilted. By increasing the distance to the object according to the scale, the tolerance for the tilt angle was improved. For example, the measurement errors (scale at 10 mm steps) when the tilt angle was −20/+20  deg at the distances of 10, 20, and 30 mm were 1.4 mm (16.3%)/−3.8  mm (27.5%), 0.3  mm(3.1%)/−1.8  mm (15.3%), and −0.1  mm (1%)/ −1.8  mm (15.3%), respectively. The endoscopic images of these conditions are shown in Fig. 5.

### 3.3 Image Evaluation of Polyp Size Estimation by Endoscopists

**Fig. 6:**
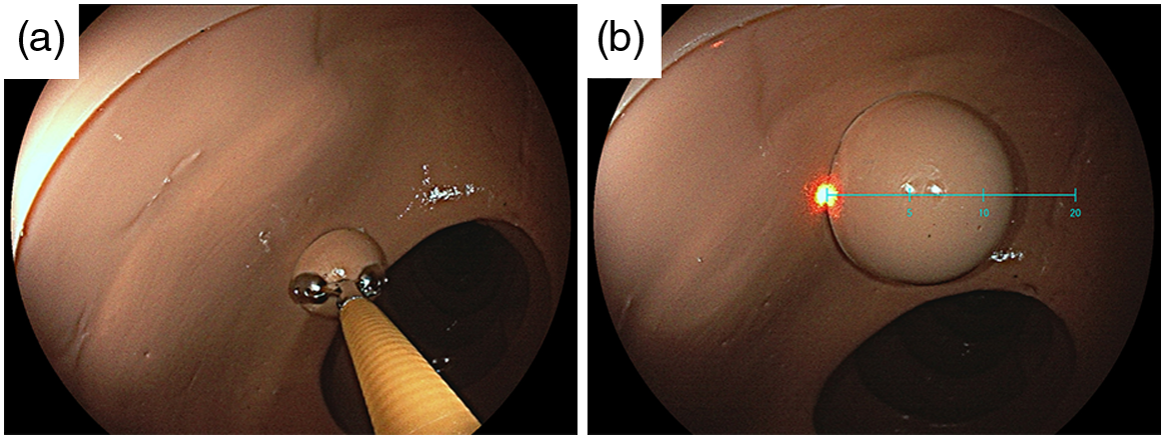
Image samples for the polyp size estimation test. (a) Polyp with a size of 10 mm with the use of forceps, and (b) polyp with a size of 15 mm estimated with the use of the virtual scale.

Three endoscopists estimated the polyp size for 33 pseudo-polyps images with the use of forceps and 33 images with the use of a virtual scale (Fig. 6). All evaluators were expert physicians who were certified as specialized endoscopists by the Japan Gastroenterological Endoscopy Society. In total, 99 estimated results for each method were analyzed.

The number of images (rates) that were correctly estimated, overestimated, and underestimated using forceps and using the virtual scale were 39 (39.4%) and 60 (60.8%), 18 (18.2%) and 6 (6.1%), and 42 (42.4%) and 33 (33.3%), respectively. The two groups were statistically independent by the Fischer’s exact test (P=0.0028). These data are shown in Table 1.

**Table 1:** Association of image numbers (rates) of correct, over-, and under estimations from 33 images with biopsy forceps and 33 images with virtual scale by three expert endoscopists (P=0.0028, Fischer’s exact test).

Group	Underestimation	Correct estimation	Overestimation
Biopsy forceps	42 (42.4%)	39 (39.4%)	18 (18.2%)
Virtual scale	33 (33.3%)	60 (60.6%)	6 (6.1%)

**Fig. 7:**
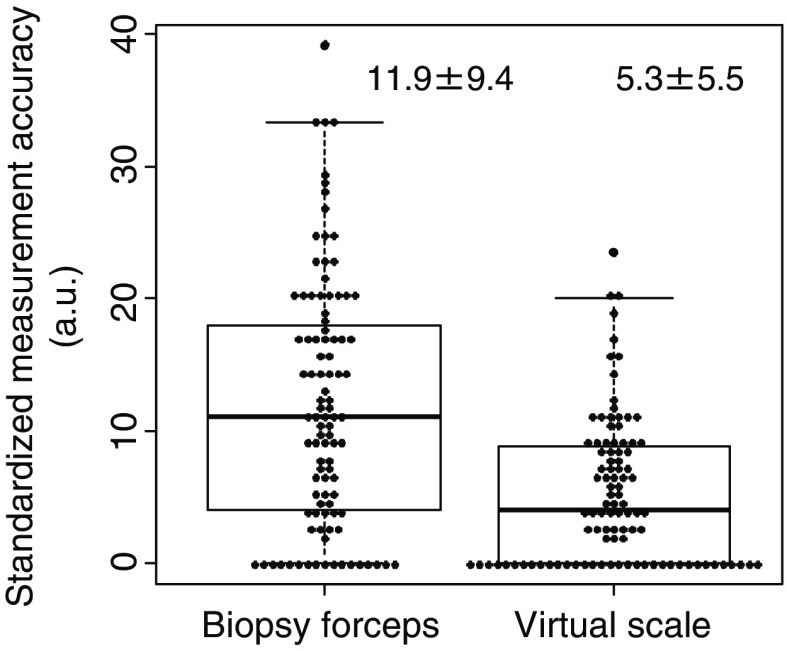
Standardized measurement accuracy from the polyp size estimation outcomes with the biopsy forceps or with virtual scale obtained from three expert endoscopists.

The standardized measurement accuracy data are shown in Fig. 7. These obtained values are 11.9%±9.4 (biopsy forceps case) and 5.3%±5.5 (virtual scale case). The results of the virtual scale showed a smaller difference from the actual size and data distribution compared with the biopsy forceps.

The standardized measurement accuracy with the virtual scale was significantly higher than that with biopsy forceps according to the Wilcoxon signed-rank test (P<0.001). For the analysis, the Wilcoxon signed-rank test was used because the difference data between biopsy forceps and virtual scale were not normally distributed.

Standardized measurement accuracy from the polyp size estimation outcomes with the biopsy forceps or with virtual scale obtained from three expert endoscopists.

## 4 Discussion

Although the estimation of polyp sizes during real-time endoscopic assessments is important for the diagnosis and choice of the treatment policy, the estimation accuracy is an issue. It is known that measurement tools, such as measurement forceps, are effective. However, a polyp size is usually estimated based only on endoscopic images in daily clinical practice because the use of additional disposable devices leads to prolonged examination times and increased costs. The lack of distance information and the characteristics of a fish-eye objective lens make it challenging to estimate the polyp size even if a digital image analysis technology is developed.

We developed a virtual scale function for the endoscope. The prototype was designed for a colonoscope so that it can be used in clinical practice in the future. This function uses a built-in laser and an image processing technology; therefore, no additional disposable device is required. It is easy to compare the size because the length of the virtual scale overlaid on the object to be measured changes adaptively in real-time on the monitor according to the distance to the object. Because it can be easily switched on and off using a button on the handle of the endoscope, it can be used as a normal colonoscope without displaying the virtual scale during the screening procedure, and the scale can be displayed only when needed without loss of inspection time. With regard to daily practice, the step of the scale was limited to three steps, namely, 5, 10, and 20 mm, which are important indicators for the treatment policy or screening examination interval so that the examination monitor would not be overloaded with information.

In this study, we demonstrated the fundamental performance of the proposed virtual scale function. The delay in adapting the suitable length of the virtual scale is sufficiently short to estimate the size in real-time. The measurement error of the virtual scale was within 0.7 mm, i.e., within the measurement range when the object was faced directly against the tip of the endoscope. The length of the virtual scale was calculated based on the assumption that the measurement target was on a plane parallel to the tip of the endoscope on which the laser beam was incident. When the measurement target is viewed from the side wall at an angle, especially when the measurement target is on the left side of the tract, the actual size is different when compared with that estimated based on the virtual scale. The change is not linear because of the distortion of the fish-eye lens. The results showed that the tolerance for the angle was improved by taking an appropriate distance depending on the size of the object to be measured. Therefore, it is recommended that the operator positions the tip of the endoscope in front of the objects to estimate the size at a distance of the medium field-of-view to achieve high accuracy with this virtual scale. When it is difficult to achieve an ideal view, understanding these characteristics may assist the estimation process.

The accuracy of the virtual scale was evaluated by expert physicians based on a pseudo-polyps model compared with the method, which used conventional biopsy forceps. The forceps estimated the size at ∼40% with an accuracy <±1  mm, and the mean measurement accuracy was 11.9%, whereas the virtual scale could accurately estimate the size at ∼60%, with a mean accuracy of 5.3%. It is known that the conventional open biopsy forceps method is more effective than the estimation without any reference devices. From the present study, the virtual scale function was expected to be as easy as the estimation process, which did not use any additional device, and was more accurate than the biopsy forceps method.

From the distribution of measurement errors, both the biopsy forceps and the virtual scale showed that there was a bias toward underestimation. It was considered that the fish-eye lens effect of the endoscope made it look smaller than the ratio of the dimensions on the image, and the point looked smaller than the reference object when the polyps were arranged diagonally. In this study, the evaluators did not know the fundamental performance results described in Sec. 3.2. Although this function has such bias, the accuracy was better than that of the conventional biopsy forceps method. It is also considered that the performance is adequate for clinical practice. The characteristics of this diagonal effect will help estimate the size accurately.

This study is associated with some limitations. The graph paper evaluation is on a flat plane, and the conditions are different from that of the gastrointestinal tract, which has a tubular structure. The tilt of the graph paper does not indicate the angle between the side walls and the endoscope because polyps, for which the size estimation is performed, are usually raised from the mucosal wall and are round in shape. In the polyp size estimation test, the objects were an artificial colon and a polyp model. The evaluation was performed only by three expert physicians in one facility. The evaluators estimated the polyp size based on only one image and without operating the endoscope. Although this evaluation method is different from that of the actual clinical setting, it is also beneficial to know the fundamental performance. First, the pseudo-polyps do not stretch or shrink, and they have fixed known sizes; the concern is about when and how to set the measured size as the gold standard because the actual mucosa changes in size as a result of intestinal peristalsis or insufflation *in vivo*, stretches according to the attachment to the pathological board after excision, or shrinks because of formalin fixation in the real world. Second, the accuracy of the size estimation was not affected by the endoscope operating skill of the endoscopist. In the usual clinical setting, endoscopists estimate the size only visually from endoscopic images. The biopsy forceps method is known to have a higher accuracy than that of visual inspection. Although there are some limitations, it is expected that the virtual scale function will have a higher accuracy than that of the current standard of care.

The prototype was implemented to be used clinically. The results discussed in this section are expected to be verified in a clinical setting.

## 5 Conclusion

A virtual scale function for endoscopy has been developed that operates in real-time and without the use of any additional devices. The accuracy of polyp size estimation using the virtual scale was significantly improved compared with that of biopsy forceps. This virtual scale function can be used to estimate polyp size easily and accurately.

## Code, Data, and Materials Availability

All data generated in this study are available within this article.
